# Medicalising diagnoses and treatment preferences: a retrospective cohort study of throat-related consultations in electronic primary care records

**DOI:** 10.3399/BJGPO.2023.0056

**Published:** 2023-11-01

**Authors:** Tom Marshall, Tom Taverner, Leila Freidoony

**Affiliations:** 1 Institute of Applied Health Research, University of Birmingham, Birmingham, UK

**Keywords:** anti-bacterial agents, antibiotics, clinician style, diagnosis, pharyngitis, primary care, referral and consultation, sore throat, tonsillitis

## Abstract

**Background:**

Rather than first diagnosing and then deciding on treatment, GPs may intuitively decide on treatment and justify this through choice of diagnosis.

**Aim:**

To investigate the relationship between choice of a medicalising diagnosis and antibiotic treatment for throat-related consultations.

**Design & setting:**

A retrospective cohort study in a large database of UK electronic primary care records between 1 January 2010 and 1 January 2020.

**Method:**

All first throat-related consultations were included, categorised as either pharyngitis/tonsillitis or sore throat. The outcome was any antibiotic prescription on the consultation date. GP-level random effects on prescribing and on diagnosis were estimated in a series of mixed-effects regression models, including age, sex, weekday, month, and clinician characteristics as fixed effects. GPs were grouped into quintiles by antibiotic prescribing propensity, and described the proportion of patients they diagnosed with pharyngitis/tonsillitis or sore throat in each quintile.

**Results:**

The analysis dataset included 393 590 throat-related consultations with 6881 staff. Diagnosis of pharyngitis/tonsillitis was strongly associated with antibiotic prescribing (adjusted odds ratio = 13.41, 95% confidence interval = 12.8 to 14.04). GP random effect accounted for 18% of variation in prescribing and for 26% of variation in diagnosis. GPs in the lowest quintile of antibiotic prescribing propensity diagnosed pharyngitis/tonsillitis on 31% of occasions, compared with 55% in the highest quintile.

**Conclusion:**

There is substantial variation among GPs in diagnosis and treatment of throat-related problems. Preference for a medicalising diagnosis is associated with a preference for antibiotics, suggesting a common propensity to both diagnose and treat.

## How this fits in

Doctors vary in their propensity to diagnose throat-related consultations such as sore throat or pharyngitis/tonsillitis, as well as in their propensity to prescribe antibiotics. A higher propensity to prescribe antibiotics is associated with a higher propensity to assign a more medicalising diagnosis. This demonstrates a common underlying propensity to diagnose and treat. It is consistent with the notion that the same intuitive cognitive processes underlie both diagnostic and treatment decisions.

## Introduction

Clinical diagnosis and treatment decisions may use either of two cognitive processes: one intuitive and fast, and the other deliberative and slow.^1,2^ In deliberative clinical reasoning, the patient’s clinical features inform a diagnosis, which precedes and then informs a treatment decision. However, doctors have treatment preferences, and therefore, their treatment decisions may be partly intuitive.^3,4^ Individuals tend to justify their decisions through rationalisation.^5^ Doctors can justify their intuitive treatment preferences through choice of diagnosis; therefore, if treatment decisions are intuitive, their choice of diagnosis and treatment preferences would be expected to align.

A number of analyses have investigated the relationship between diagnosis and antibiotic treatment. In a US telemedicine centre, physicians in the lowest quartile for antibiotic prescribing diagnosed sinusitis (considered a stronger justification for antibiotic treatment) in 35% of upper respiratory infections, while physicians in the highest quartile diagnosed sinusitis in 59% of upper respiratory infections.^6^ An analysis of upper respiratory infections in a US outpatient setting reported equivalent figures of 4% and 33%, respectively.^7^ Similar observations were made in a single US practice.^8^ In Germany, low-antibiotic-prescribing practices diagnosed 45.2% of respiratory infections as bacterial, compared with 64.5% in high antibiotic-prescribing practices.^9^ In Canada, low-prescribing physicians diagnosed bacterial infection in 31.0% of respiratory infections compared with 65.4% in high-prescribing physicians.^10^ In the Netherlands, choice of diagnosis at the practice level explained a substantial part of variation in practice antibiotic prescribing.^11^ These analyses varied in the extent to which they adjusted for potential confounders, but the findings were consistent across different settings. Primary care physicians have measurable underlying preferences for intervention, which are associated with their antibiotic prescribing behaviour.^12^ There is also evidence that intuitive (automatic) processes play a part in treatment decisions.^13,14^

Throat-related consultations are common, and in the UK most result in an antibiotic prescription.^15^ Patients typically present with a symptom of throat pain, which may be assigned a diagnosis of infection, or simply described as sore throat. A diagnosis of bacterial (particularly streptococcal) infection justifies antibiotic prescription.^16^ Point-of-care tests for bacterial throat infection are not recommended for general use in UK primary care.^17^ While symptom scores help to distinguish bacterial from non-bacterial sore throats, high scores only weakly predict bacterial infection; symptom elicitation is subjective, and as such, symptom scores are infrequently used in practice.^18 –21^ Choice of diagnosis for throat-related symptoms is therefore mainly a matter of clinical judgment.

Choice of diagnosis and treatment decisions were investigated, using the example of consultations for throat-related consultations and antibiotic prescribing. It is hypothesised that GPs have an underlying preference both for antibiotic treatment and for choice of diagnosis. It is predicted, therefore, that propensity to prescribe antibiotics and propensity to choose a medicalising diagnosis justifying antibiotics will be correlated.

## Method

### Data source and study cohort

This is an open cohort study of patients registered with general practices contributing to IMRD (IQVIA Medical Research Database), a database of anonymised electronic patient records from over 688 UK general practices.^22^ The database is broadly generalisable to the UK population in terms of demographics and medical condition prevalence, although some regions (for example, Scotland) are over-represented.^23,24^ It contains clinically coded information on diagnoses, symptoms, and treatments. All research using anonymised patient records from The Health Improvement Network (THIN) has prior approval from the NHS South East Multi-centre Research Ethics Committee subject to independent scientific review.^25^

The researchers had complete access to the full database. Data were only included after the date of practice acceptable mortality reporting (the date after which patient deregistrations were recorded consistently), ensuring the registered population was accurate.^26^ Patients of all ages were eligible for inclusion if they had been registered for at least 12 months between 1 January 2010 and 1 January 2020, and had a clinical code indicating a throat-related consultation (Supplementary Table 1). Data extraction was undertaken using the data extraction for epidemiological research (DExTER) tool.^27^

The study is reported in accordance with the REporting of studies Conducted using Observational Routinely-collected Data (RECORD) guidelines, and a completed checklist is provided as a supplementary document.^28^

### Study design

A retrospective cohort study was carried out on all first episodes of throat-related consultations with patients of any age. The outcome was a prescription of any antibiotic (identified as any drug in the *British National Formulary* chapter 5.1)^29^ on the same date as the throat-related consultation.

Consultations in the dataset are assigned a clinical code from a comprehensive list of clinical terms which has been in use in the UK since 1985 (Read code version 2).^30^ Throat-related consultations assigned clinical codes specifying chronic infection were excluded, as these codes imply that this was not a first episode (excluded codes are listed in Supplementary Table 2). Clinical codes were categorised into the two groups likely to be clinically associated with probability of antibiotic prescription: a more medicalising diagnosis of pharyngitis/tonsillitis (a clinical code indicating pharyngitis, throat infection, bacterial infection, tonsillitis, or abscess), or sore throat (a clinical code describing a symptom of throat pain) (Supplementary Table 1).

### Predictor variables and covariates

Both diagnosis of sore throat and antibiotic prescribing are influenced by the patient’s age and sex; therefore, these are included as covariates in the null model.^19,31,32^ Age was grouped into bands to allow for non-linear effects of age (0–4, 5–9, 10–19, 20–29, 30–39, 40–49, 50–59, 60–69, 70–79, 80–100 years of age). As weekday and season affect antibiotic prescribing, day of week and month were included.^33,34^

Previous research has shown the clinician’s gender affects antibiotic prescribing decisions, and the type of clinician consulted (for example, GP partner, salaried GP, locum GP, or practice nurse) might affect diagnosis and prescribing, as it is a proxy for clinical experience.^35^ GP partners are owners of general practices and employ other staff members, including salaried GPs. For simplicity, in this article all of these clinician types are referred to as GP, although a small minority were not GPs.

### Analysis

Diagnosis and prescribing occur at the level of the individual patient, and are likely to be influenced by the individual patient’s characteristics (for example, age and sex) and by characteristics of the GP across multiple patients. GP characteristics may be known (for example, clinical role and gender) or unmeasured (propensity to diagnose and to prescribe antibiotics). Multilevel models take account of the effects of patient characteristics and known GP characteristics as fixed effects, with a random effect reflecting varying average rates of diagnosis and prescribing between GPs.^36^ The modelling strategy used was to test the significance of GP-level independent variables in a series of mixed-effects regression models, fit using the maximum likelihood (Laplace approximation) method (glmer in the R package lme4).

**Figure 1.**
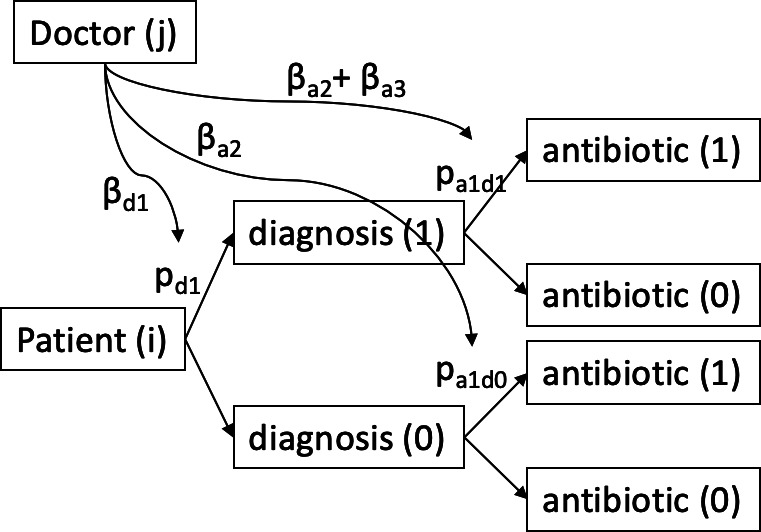
Possible pathways for GP-level influence on diagnosis and prescription.

The sequence of possible GP influences on diagnosis and prescription is conceptualised in Figure 1. The patient has an underlying probability of receiving a diagnosis of pharyngitis/tonsillitis (p_d1_), which may be influenced by the GP (propensity to diagnose). Once diagnosed, the probability of antibiotic prescription for patients with (p_a1d1_) and without (p_a1d0_) a diagnosis of pharyngitis/tonsillitis may be influenced by the GP (propensity to prescribe). The probability of antibiotic prescription for a specific diagnosis should be thought of as a uniform GP-level random intercept for all patients, with a possibility of a random slope; that is, an effect modification between GPs of the probability of antibiotic prescription for a given diagnosis.

The following questions were investigated in order: if GPs have a propensity to diagnose; if GPs have a propensity to prescribe antibiotics, with and without diagnosis as mediator; and if GPs have a propensity to prescribe with an interaction between propensity to diagnose and propensity to prescribe.

### GPs’ propensity to diagnose

Coding sore throat as 0 and pharyngitis/tonsillitis as 1, diagnosis was modelled as a function of the null model with GP identifier (staffid) as a random intercept. The variance for the GP identifier (staffid) after adjustment for covariates represents the variability between different GPs’ propensity to diagnose. The models were:

(1a) logit(p(diagnosis)) ~ (patient covariates), a generalised linear model for diagnosis as a function of age, sex, weekday, and month.

(1b) logit(p(diagnosis)) ~ (patient covariates) + (1|staffid), testing a GP-level random effect on diagnosis. The variance for the GP identifier (staffid) after adjustment for covariates represents the variability between different GPs’ propensity to diagnose (pharyngitis/tonsillitis or sore throat). Model 1b can be represented as:

$$logit(p_{d1}(i,j))=\beta_{d0}+\beta_{d1}(j);\;\beta_{d1}(j)∼N(0,\sigma_{d}^{2})$$

Comparing model 1a with 1b allows a test of the size of the GP effect or GP-level variance in diagnosis, β_d1_(j).

### GPs’ propensity to prescribe antibiotics

In a similar way, antibiotic prescribing was modelled as a function of the null model (2a), including diagnosis (2b) and then with GP identifier (staffid) as a random intercept, without including diagnosis (2c) and including diagnosis (2d). The adjusted variance for staffid represents the variability between different GPs’ propensity to prescribe.

(2b) logit(p(antibiotic)) ~ (patient covariates) + diagnosis, is a generalised linear model for antibiotic prescribing as a function of patient-level covariates (age, sex, weekday, and month).

(2c) logit(p(antibiotic)) ~ (patient covariates) + (1|staffid) models a GP-level random effect on antibiotic prescribing without taking account of diagnosis. This is related to the probabilities of prescription for patient (i), GP (j) p_a1d0_, and p_a1d1_ in Figure 1 via the relations:

$$logit(p_{a1d0})=\beta_{a0}+\beta_{a2}(j);\;logit(p_{a1d1})=\beta_{a0}+\beta_{a1}+\beta_{a2}(j)+\beta_{a3}(j)$$

(2d) logit(p(antibiotic)) ~ (patient covariates) + diagnosis + (1|staffid), testing a GP-level random intercept in antibiotic prescription; that is, the tendency of a GP to prescribe antibiotic depends on the GP. The variance for the GP identifier (staffid) after adjustment for covariates represents the variability between different GPs’ propensity to prescribe antibiotics. Model 2b can be represented as

$$logit(p_{a1}(i,j))=\beta_{a0}+\beta_{a1}d(i)+\beta_{a2}(j);\;\beta_{a2}(j)∼N(0,\sigma_{a}^{2})$$

Comparing model 2b and 2d allows a test of GP-level variance in antibiotic prescription, β_a2_(j).

### GPs’ propensity to prescribe antibiotics with an interaction between diagnosis and prescribing propensities

A final model (2e) included an interaction term between GPs’ propensity to prescribe antibiotics and diagnosis. (2e) logit(p(antibiotic)) ~ (patient covariates) + diagnosis + (1 + diagnosis|staffid), a model with a GP-level random intercept and interaction in antibiotic prescription; that is, the tendency of a GP to prescribe antibiotic depends on the GP, and there is also variation among GPs in how readily they prescribe in the presence of pharyngitis/tonsillitis or sore throat. Moreover, a correlation coefficient between the random-effects intercept and slope represents the relationship between GP-level variability in prescription and GP-level variability in the diagnosis. This correlation may be positive, negative, or zero:

$$logit(p{)}_{a1}(i,j))=\beta_{a0}+\beta_{a1}d(i)+\beta_{a2}(j)+\beta_{a3}(j)d(i);\;\left(\beta_{a2}(j),\beta_{a3}(j)\right)∼N(0,\Sigma_{a})$$

Comparing models 2c, 2d, and 2e allows a hypothesis test for GP-level variance in the interaction between diagnosis and antibiotic prescription. This indicates if the interaction term improves the model fit.

For the series of models (1a-1b, 2b-2b, 2b-2e), model fits were compared and *P*-values generated for the hypothesis that each successively added random effect had variance of exactly 0, by means of the generalised likelihood ratio test, and by recording Akaike information criterion (AIC). The intraclass coefficient of correlation ρ was also calculated, which is the proportion of variance of the group level (here, inter-GP variation) to the total variance.^37^

$$\rho =var(u)/(var(u)+\pi^{2}/3)$$

The proportion of variability in prescribing explained by the random (staff-level) effects was explored using the method of Nakagawa and Shielzeth.^38^ These authors present two calculations for a mixed-effects model R^2^: marginal (R^2^m) and conditional (R^2^c). R^2^m is concerned with variance explained by fixed effects, and conditional R^2^c is concerned with variance explained by both fixed and random effects. The difference (R^2^c – R^2^m) reflects how much variability is explained by random effects, and can give insights into data. This model has been extended to the case of random-effects models with random slopes, and these extensions been incorporated into the r.squaredGLMM function in the MuMIn package.^39,40^ This was used to calculate R^2^c and R^2^m for the random-effects models.

The coefficient for the GP identifier (staffid) after adjustment for covariates is the GP’s propensity to prescribe antibiotics. Correlation coefficients for GP’s propensity to diagnose (pharyngitis/tonsillitis or sore throat) and GP’s propensity to prescribe antibiotics were calculated. To illustrate the relationship between propensity to prescribe and propensity to diagnose, GPs were categorised into quintiles by propensity to prescribe antibiotics, and the proportion of diagnoses in each category (pharyngitis/tonsillitis or sore throat) were determined for each quintile.

## Results

### Data preparation

**Figure 2.**
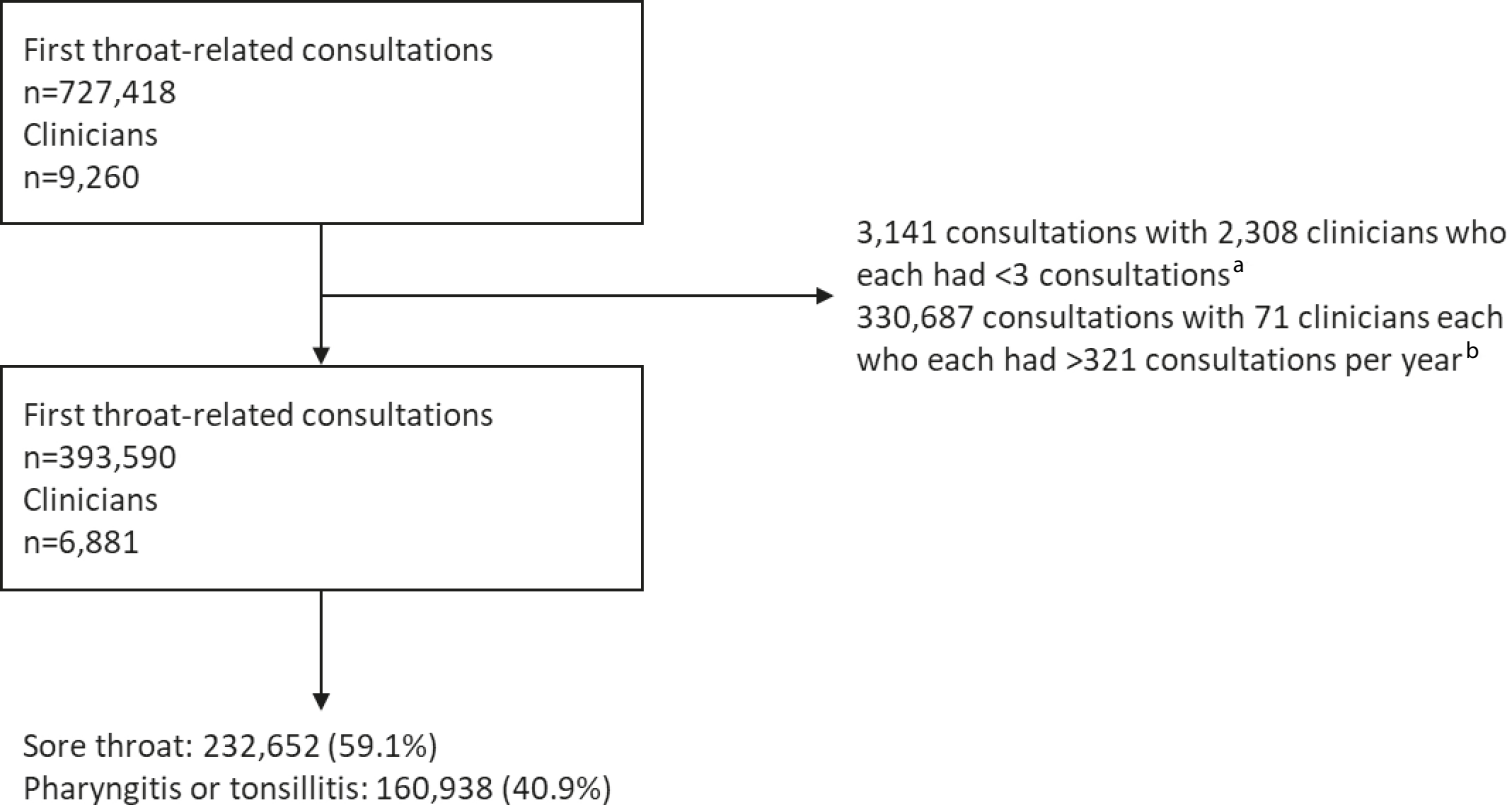
Selection of patient for inclusion in the analysis. ^a^Too few consultations per clinician. ^b^Implausibly high numbers of consultations per clinician.

A total of 727 418 first throat-related consultations were identified, attributed to 9260 clinicians. However, 3141 consultations with 2308 clinicians were removed as they had <3 consultations, which was considered too few to contribute substantially to the random effects and caused convergence problems. Also removed were 330 687 throat-related consultations attributed to 71 staff identifiers (staffid), because the implausibly high number (>321) of yearly consultations for sore throat was suggested that the same staff identifier was used by multiple clinicians (see Supplementary Table 3 for rationale for upper limit). This left an analysis dataset of 393 590 first throat-related consultations with 6881 staff: 59.1% were diagnosed as sore throat and 40.9% as pharyngitis/tonsillitis (Figure 2).

### Descriptive analyses

A diagnosis of pharyngitis/tonsillitis was more common in younger age groups. Antibiotics were prescribed for 57.0% of consultations: 38.5% of sore throat consultations and 83.8% of pharyngitis/tonsillitis consultations (Table 1). The most commonly prescribed antibiotics were phenoxymethylpenicillin (67.0%), amoxicillin (17.0%), erythromycin (7.5%), and clarithromycin (5.2%).

**Table 1.** Description of the study cohort

	Sore throat		Pharyngitis or tonsillitis	
*N* (% of total)	232 652 (59.1)		160 938 (40.9)	
	*n*	%	*n*	%
Antibiotics	89 649	38.5	134 811	83.8
Age band, years				
0–4	18 086	7.8	44 632	27.7
5–9	24 069	10.3	26 379	16.4
10–19	33 133	14.2	24 783	15.4
20–29	27 188	11.7	19 222	11.9
30–39	36 341	15.6	21 058	13.1
40–49	30 003	12.9	11 775	7.3
50–59	24 675	10.6	6591	4.1
60–69	20 481	8.8	3984	2.5
70–79	12 534	5.4	1770	1.1
80–101	6142	2.6	744	0.5
Sex				
Male	95 855	41.2	72 968	45.3
Female	136 797	58.8	87 970	54.7

### Multilevel analysis

#### GPs’ propensity to diagnose

Compared with the null model for diagnosis (Model 1a), the model including a heterogeneous term for GP effect (Model 1b) is a significantly better fit, with an intraclass coefficient (ρ=0.26) indicating the difference between GPs’ accounts for one-quarter of variation in diagnosis. (Table 2).

**Table 2.** Random-effects models investigated for prediction of diagnosis (pharyngitis/tonsillitis or sore throat) and antibiotic prescribing

Models predicting diagnosis	AIC	*P* value	Variance ofrandom effect	OR for diagnosis(95% CI)	% variance attributable to GP	Marginal R^2^, %	Conditional R^2^, %	R^2^c – R^2^m, %
1a Null model for diagnosis diagnosis ~	477462.9	NA	NA	NA		NA	NA	
1b Null model + GP random-effect diagnosis ~ null model + (1|staffid)	441144.2	<0.001	1.16	NA	26.0	13.1	32.3	19.2

Models predicting antibiotic prescribing	AIC	*P* value	Variance ofrandom effect	OR for antibioticprescribing (95% CI)	% varianceattributable to GP	MarginalR^2^, %	ConditionalR^2^, %	R^2^c – R^2^m,%
Null model for antibiotic abx ~ null model	515680.4	NA	NA	NA		NA	NA	
2b Diagnosis-only model for antibiotic abx ~ diagnosis + null model	437269.3	<0.001	NA	2.24 (2.22 to 2.26)		NA	NA	
2c GP random-effect only model for antibiotic abx ~ null model + (1|staffid)	496961.3	<0.001	0.59	NA	15	5.8	17.7	11.9
2d and GP random-intercept model for antibiotic abx ~ diagnosis + null model + (1|staffid)	417323.6	<0.001	0.81	2.48 (2.46 to 2.50)	20	26.3	38.6	12.3
2e and GP random-slope model for antibiotic abx ~ diagnosis + null model + (1 + diagnosis|staffid)	412008.4	<0.001	0.73	2.60 (2.55 to 2.64)	18	26.3	42.7	16.4

a Null model includes patient age (in age bands), patient sex, day of week, month, clinician type (for example, GP partner, salaried GP, locum GP, practice nurse), clinician sex. *P*-values from likelihood ratio test. Marginal R^2^ = variance explained by fixed effects. (Conditional R^2^ – Marginal R^2^) = variance explained by random effects. AIC = Akaika information criterion. abx = antibiotic prescribing. CI = confidence intervals. GP = clinician (includes a small number of practice nurse consultations). NA = not available. OR = odds ratio.

A diagnosis of pharyngitis/tonsillitis is much more common in young children. With age 40–50 years as the reference category, the adjusted odds ratio (aOR) for age 0–4 years is 6.57 (95% confidence interval [CI] = 6.38 to 6.77), and the aOR declines with age. A diagnosis of pharyngitis/tonsillitis is also more likely at weekends: with Monday as the reference category, Sunday’s aOR is 2.33 (95% CI = 2.13 to 2.55). The choice of reference categories was arbitrary. A diagnosis of pharyngitis/tonsillitis is less likely if a practice nurse is consulted than if a GP partner is (aOR = 0.57, 95% CI = 0.55 to 0.59). Furthermore, a diagnosis of pharyngitis/tonsillitis is slightly less likely if a female clinician is consulted, and is less likely in September and October than it is in January. Supplementary Table 4 shows the fixed-effect coefficients for a diagnosis of pharyngitis/tonsillitis.

#### GPs’ propensity to prescribe antibiotics

Compared with the null model for antibiotic prescribing, diagnosis is a significant predictor of prescribing (Model 2b) and a GP random effect is a significant term (Model 2c) (Table 2). Including both diagnosis and a GP random effect, variation between GPs is a highly significant term (Model 2d vs 2b), and accounts for 20% of variability in antibiotic prescription. Model 2e, which allows the effect of GP on antibiotic prescription to vary by diagnosis, is even more highly favoured (*P*<0.001 for 2e vs 2b, ρ=0.20). This model explains 42.7% of the variance in antibiotic prescription, and 18% of the variance in antibiotic prescription is attributable to the GP.

Supplementary Table 5 shows the fixed-effect coefficients for prescription of an antibiotic, including the effect of diagnosis, and a GP random effect (Model 2d). As expected, prescribing an antibiotic is strongly associated with a diagnosis of pharyngitis/tonsillitis (aOR = 11.95, 95% CI = 11.72 to 12.19). Antibiotic prescribing is also slightly likelier if the consultation is with a locum GP (aOR = 1.17, 95% CI = 1.14 to 1.21). Antibiotic prescribing is much less common at the weekend, compared with Monday (Sunday aOR = 0.10, 95% CI = 0.09 to 0.11). Compared with consultations with a GP partner, antibiotic prescribing is less likely if a GP senior partner (aOR = 0.85, 95% CI = 0.76 to 0.93) or ‘other’ category of staff (aOR = 0.32, 95% CI = 0.31 to 0.33) is consulted.

### Relationship between propensity to prescribe and propensity to diagnose

**Figure 3.**
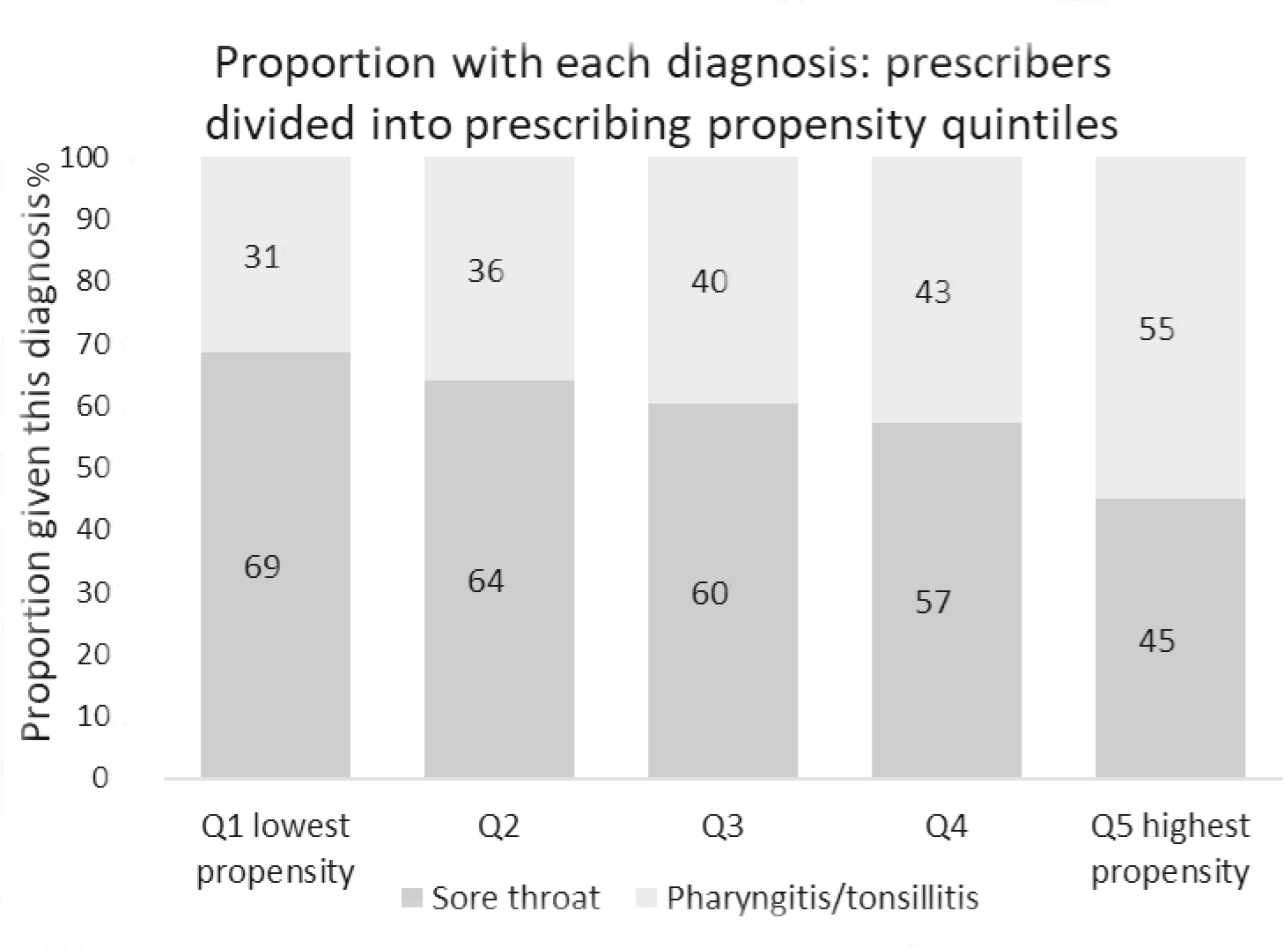
Relationship between propensity to prescribe antibiotics and GP choice of diagnosis. Q = quintile.

There was a clear relationship between propensity to diagnose and propensity to prescribe antibiotics. GPs in the lowest prescribing propensity quintile diagnosed pharyngitis/tonsillitis on 31% of occasions, compared with 55% in the highest prescribing propensity quintile (Figure 3). The lowest prescribing propensity quintile prescribed antibiotics for 25% of sore throat diagnoses and 64% of pharyngitis/tonsillitis diagnoses, compared with 57% and 94% for the highest quintile, respectively (Supplementary Table 6).

## Discussion

### Summary

After adjusting for patient and temporal characteristics, variation between individual GPs accounts for about one-quarter of the variation in diagnosis of first throat-related consultations. As expected, a medicalising diagnosis of pharyngitis/tonsillitis is strongly associated with antibiotic prescription. There is also variation between individual GPs in antibiotic prescribing, which is partly mediated through GP choice of diagnosis. GPs with a higher propensity to prescribe antibiotics also have a higher propensity to diagnose pharyngitis/tonsillitis.

### Strengths and limitations

This is a large dataset and reflects usual primary care. Prescription data are well captured, and it is likely that antibiotic prescriptions issued on the same day as a throat-related consultations are for that indication. The binary categorisation of diagnoses is a simplification of a continuum of sore throat severity and did not use information in free text, which may have led to some miscategorisation. The observed strong relationship between diagnosis and antibiotic prescribing, however, suggests the categorisation has face validity. Some staff identifiers that were used by multiple clinicians may have been included, which would reduce between-GP variation. Some staff who could record a diagnosis but could not prescribe may also have been included (in the other staff and practice nurse categories), which would weaken the relationship between propensity to diagnose and to prescribe. Unusually low antibiotic prescribing at weekends may reflect under-recording of prescriptions issued out-of-hours.

Not every possible confounder which might influence prescribing was included; if there are systematic differences in patients seen by different GPs, this could account for some of the GP’s apparent propensity to prescribe. One potentially important omission is the presence of patient comorbidities, which has been associated both with higher and lower antibiotic prescribing in different settings.^9,31^ Clustering of patients with comorbidities by GP could account for some of the GP’s propensity to prescribe. However, age was included, which is strongly linked to comorbidity and is more likely to be associated with GP than comorbidity as such. Practice characteristics such as size or rurality were not included, nor was the general practice considered as an independent random effect. Other studies of prescribing have identified a practice-level effect, but also observed the GP effect to be much greater.^41^

### Comparison with existing literature

This study’s findings are consistent with other research that clinicians’ propensity to prescribe antibiotics is partly mediated through a related propensity to diagnose. This was first suggested over half a century ago.^42^ In US primary care, this manifested as a physician propensity to diagnose upper respiratory infection as sinusitis.^6–8^ In other primary care settings, physicians showed a propensity to diagnose bacterial infection, which is linked to a propensity to prescribe antibiotics.^9–11^

### Implications for research and practice

This analysis finds support for an underlying clinician propensity to use a more medicalising diagnosis, and their propensity to prescribe antibiotics (for either diagnosis). It remains undetermined whether clinician propensity is an individual preference (practice style) or caused by contextual factors (time pressure, practice norms, relational continuity of care). The role of individual factors could be further explored by measuring individual clinician preference;^12^ the role of context could be explored by investigating clustering of clinician propensities at the level of general practice.

These results have implications for understanding both diagnosis and prescribing. As choice of diagnosis is not independent of prescribing propensity, adjusting prescribing rates for diagnosis will reduce apparent clinician variation. Choice of diagnostic label is itself important, because it influences patients’ expectations about management and treatment.^43,44^ If choice of diagnosis also cognitively enables clinicians to treat, changing diagnostic language may be a way of changing clinicians’ prescribing behaviour.

The greater the role of clinician judgment in diagnosis, the greater the potential for diagnostic preference to mediate treatment preference. Aside from throat-related consultations, choice of diagnosis may also mediate prescribing of other drugs where there is substantial between-practice variation; for example, proton-pump inhibitors, benzodiazepines, or antidepressants.^45–47^ Practice-level and GP-level prescribing rates correlate across different drug classes.^48,49^ If diagnostic preferences also correlate across different types of presenting problems, this would lend support to the hypothesis of distinctive practice styles.

## Public and patient involvement

Patients and members of the public were not involved in the design of this study or the development of the research question.

## Data

The full dataset will be made available to researchers on request from the corresponding author. Individual consent was not obtained, but the presented data are anonymised and risk of identification is low.
